# Impact of Subsidies and Socioeconomic Status on Varicella Vaccination in Greater Tokyo, Japan

**DOI:** 10.3389/fped.2016.00019

**Published:** 2016-03-15

**Authors:** Kei Nagaoka, Takeo Fujiwara

**Affiliations:** ^1^Department of Social Medicine, National Research Institute for Child Health and Development, Tokyo, Japan

**Keywords:** varicella vaccine, vaccine subsidy, varicella, socioeconomic status

## Abstract

**Background:**

Although the control of varicella outbreaks is an important health issue, cost could present a major barrier for vaccination. The aim of this study is to investigate the association of vaccine subsidies and caregivers’ socioeconomic status with varicella vaccine coverage of their children in Greater Tokyo, Japan, before the period that varicella vaccination was included in routine immunization program.

**Methods:**

Participants were recruited from two different cities. In Chiba city, parents of 18-month-old infants (*N* = 378) undergoing a medical examination in July 2013 were recruited at a clinic where no subsidy for varicella immunization was provided. In Nishitokyo city, parents of 24- to 30-month-old children (*N* = 315) undergoing a health checkup in July and August 2013 were recruited at a clinic where a partial subsidy was provided. The association between household income and varicella immunization was investigated by multivariate logistic regression stratified by city.

**Results:**

Vaccine coverage was 61.0% in Chiba city and 73.3% in Nishitokyo city. In Chiba city, odds ratios of middle and high household income for varicella immunization were 4.22 [95% confidence interval (CI): 1.65–10.7] and 5.94 (95% CI: 1.89–18.6), respectively, compared to low household income. However, household income was not associated with varicella vaccination in Nishitokyo city. Neither working status nor education was associated with vaccination in both cities.

**Conclusion:**

While household income was associated with high vaccine coverage in the city with no vaccine subsidy, this association was not observed in the city where the subsidy was given, which suggests that cost is a barrier for varicella immunization. Thus, in countries where varicella vaccination is not included in routine immunization programs, introducing subsidies nationwide or routine immunization programs for varicella vaccination would be an important approach to eliminate inequality in vaccine coverage.

## Introduction

Varicella is a common, highly infectious disease caused by the varicella-zoster virus that mainly affects young children. Although the natural course of the disease is self-limiting, it lasts 4–5 days (1). Until the rash disappears, affected children are not allowed to go to nursery, kindergarten, and school, and parents need to stay at home to take care of them, which increases the social cost of varicella infection. An estimated one million children are diagnosed with varicella each year in Japan, and of this number at least 4,000 children require admission to hospital. Although deaths are rare, varicella can be fatal, especially in neonates and immunocompromised individuals; 20 patients have died due to complications of varicella in Japan (2). Needless to say, medical costs associated with varicella infection are a huge burden. The total annual cost associated with varicella is estimated to be JPY 52.2 billion (3). Antiviral drugs are effective for shortening the duration of the disease (4) and are used in many cases in Japan, even though the patients have no risk of being severe cases, which induces higher cost.

To avoid these burdens, varicella vaccination was included as part of routine immunization programs in October 2014. Before this inclusion, children in Japan only received varicella vaccination if their caregivers elected to do so; however, their caregivers must cover the cost of the vaccination (approximately 100 USD). According to the best available data, varicella vaccination coverage was estimated to be as low as 30% (5), suggesting that cost was a possible barrier for varicella vaccination. The impact of immunization cost may exist in addition to previously reported risk factors, including poverty (6–9), single parenthood (6, 8, 9), ethnic minorities (8, 10–12), young maternal age (<30 years old), maternal working status, subsequent child, interaction with other children, lack of knowledge on vaccination, a high concern about adverse effects of vaccination (13), or living in the inner city (14). Thus, it is important to show the direct effect of vaccination subsidies on varicella vaccination for other countries where varicella vaccination is not included in routine immunization program, and people have to rely on subsidies to get vaccinated against varicella.

In Japan, before the introduction of varicella routine immunization program, some local municipalities offered subsidies for varicella vaccination to caregivers, which provided a valuable opportunity to investigate the impact of subsidies on varicella vaccination. Furthermore, we can assess the impact of the vaccine subsidy on varicella vaccination in conjunction with socioeconomic status of the household (e.g., household income or education). A previous study showed a slight increase in varicella vaccine coverage after the introduction of a partial subsidy in two cities in Japan (15). However, the study was conducted in rural cities where the vaccine coverage was lower than urban cities, and it did not investigate the impact of socioeconomic status on vaccine coverage.

Thus, the aim of this study is to elucidate the impact of a vaccine subsidy and caregivers’ socioeconomic status on varicella vaccination.

## Materials and Methods

Participants were recruited from two cities: 571 parents who attended medical examinations for their 18-month-old infants in Chiba city in July 2013 were recruited at a clinic with no subsidy for varicella immunization, and 342 parents who attended health consultations for their 24- to 30-month-old children in Nishitokyo city in July and September 2013 were recruited at a clinic with a partial subsidy. In both cities, all mothers who attended the event were recruited to answer a questionnaire. It cost JPY 5,000–10,000 (almost 50–100 USD) to vaccinate their child depending on the clinic in Chiba city, whereas it cost JPY 4,000 (almost 40 USD) in Nishitokyo city. Both Chiba and Nishitokyo cities are districts within Greater Tokyo. In August 2013, the population in Chiba and Nishitokyo cities was 963,985 and 197,703, respectively. The number of 1-year-old children in Chiba and Nishitokyo cities was 8,257 and 1,777, respectively. Our study was approved by the ethics committee of the National Center for Child Health and Development (No. 668), which determined it was unnecessary to obtain consent from participants given that responding to the anonymous questionnaire already implied consent to participate in the study.

We prepared a questionnaire that asked participants whether they vaccinated their children, the reasons for not vaccinating their children, as well as questions on demographics and socioeconomic status (parental education and annual household income).

We employed a multiple logistic regression model to determine the odds ratio (OR) of socioeconomic status for varicella immunization, stratified by cities. Furthermore, the interaction effect between the city and the household income on vaccination was also estimated. We used STATA SE statistical package, version 11 (StataCorp., College Station, TX, USA) for statistical analysis.

## Results

### Descriptive Statistics

In July 2013, 571 children aged 18 months were living in Chiba city. Of this number, 535 (93.7%) attended medical examinations with a parent and 378 (66.2%) parents completed the questionnaires. In July and August 2013, 342 children aged between 24 and 30 months attended health consultations with a parent. Of this number, 315 (92.1%) parents completed the questionnaires. Demographics and socioeconomic status for Chiba and Nishitokyo cities are shown in Table 1. The proportion of parents with more than two children was higher in Chiba city than Nishitokyo city (13.3 vs. 6.2%, *p* < 0.001). Although the age of children was older in Nishitokyo city than Chiba city by 6 months or more, parents’ socioeconomic status was almost similar in both cities. However, one slight difference in socioeconomic status was found: more parents in Chiba city had full-time jobs than those in Nishitokyo city (27.4 vs. 17.7%, *p* = 0.007), and fewer parents in Chiba city had an undergraduate degree compared to Nishitokyo city (36.4 vs. 49.4%, *p* < 0.001). The proportion of parents whose annual household income was lower than JPY two million was slightly higher in Chiba city than in Nishitokyo city, although the difference was not statistically significant (3.8 vs. 1.4%, *p* = 0.062).

**Table 1:** **Demographics of Chiba and Nishitokyo cities**.

	Chiba city (*N* = 372)	Nishitokyo city (*N* = 314)	*p*-Value
Age of the caregiver	33.1 ± 4.8 (21–46)	34.1 ± 4.5 (21–46)	0.009
Sex (F/M ratio)	1.04	0.87	0.26
Birth weight (g)	3,012 ± 444 (485–4,206)	2,984 ± 405 (1,214–4,006)	0.40
Gestational age (weeks)	38.6 ± 1.8 (27–41)	38.7 ± 2.2 (28–42)	0.91
Birth order			0.005
First	177 (50%)	165 (53.9%)	
Second	130 (36.7%)	122 (39.9%)	
Third	44 (12.4%)	13 (4.3%)	
>Fourth	3 (0.9%)	6 (1.9%)	
Past history			
Febrile seizure	19 (5.0%)	17 (5.4%)	0.83
Food allergy	48 (12.7%)	37 (11.8%)	0.70
Atopic dermatitis	25 (6.6%)	11 (3.5%)	0.065
Bronchial asthma	19 (5.0%)	8 (2.5%)	0.092
Others	17 (4.5%)	11 (3.5%)	0.50
No conditions	242 (64.0%)	215 (68.3%)	0.24
Measles vaccine coverage	341 (97.4%)	286 (97.3%)	0.91
Number of family members	3.7 ± 0.9 (2–7)	3.7 ± 0.9 (2–8)	0.77
Working status			0.012
Full time	97 (27.4%)	54 (17.7%)	
Part time	40 (11.3%)	39 (12.8%)	
Not working	217 (61.3%)	213 (69.6%)	
Education			0.022
Junior high school	10 (2.8%)	6 (2.0%)	
High school	69 (19.6%)	52 (17.1%)	
Junior college	75 (21.3%)	53 (17.4%)	
Vocational college	67 (19.0%)	43 (14.1%)	
University	115 (32.7%)	140 (46.1%)	
Graduate school	13 (3.7%)	10 (3.3%)	
Annual household income (JPY)			0.30
Less than 2 million	13 (3.8%)	4 (1.4%)	
2–4 million	63 (18.2%)	61 (20.8%)	
4–6 million	98 (28.3%)	89 (30.4%)	
6–8 million	82 (23.7%)	67 (22.9%)	
8–10 million	38 (11.0%)	40 (13.7%)	
More than 10 million	29 (8.4%)	17 (5.8%)	
Do not wish to answer	23 (6.7%)	15 (5.1%)	

### Varicella Vaccine Coverage

Varicella vaccine coverage in Chiba and Nishitokyo cities was 61.0 and 73.3%, respectively (Table 2). Coverage increased to 65.6% in Chiba city and 78.5% in Nishitokyo city when we excluded those children who were not vaccinated due to a past varicella infection or other medical reasons. We employed the latter coverage as dependent variables.

**Table 2:** **Varicella vaccine coverage rate**.

	Chiba city	Nishitokyo city
Coverage rate	227/372 (61.0%)	230/314 (73.3%)
Coverage rate (children not immunized due to previous varicella infection excluded)	227/346 (65.6%)	230/293 (78.5%)

### Association between Socioeconomic Status and Varicella Vaccination

The association between socioeconomic status and varicella vaccination is shown in Table 3. In our bivariate model, education and household income in Chiba city were positively associated with varicella vaccination. Similarly, in Nishitokyo city, education and household income were positively associated with varicella vaccination, but the magnitude was lesser.

**Table 3:** **Univariate analysis of association between varicella vaccination and variables of participants**.

	Chiba city OR (95% CI)	Nishitokyo city OR (95% CI)
Age of caregivers (compared to 20s)		
30s	**3.57 (2.06–6.21)**	**2.11 (1.01–4.41)**
40s	**6.92 (2.40–20.0)**	2.62 (0.92–7.41)
Female (compared to male)	0.84 (0.53–1.33)	1.14 (0.65–2.01)
Birth weight <2,500 g	0.81 (0.38–1.72)	1.16 (0.42–3.22)
Period of gestation <37 weeks	2.05 (0.67–6.26)	0.67 (0.26–1.68)
Birth order (as one child increases)	**0.54 (0.39–0.74)**	0.79 (0.54–1.15)
Past history		
Febrile seizure	0.96 (0.35–2.66)	0.58 (0.19–1.74)
Food allergy	1.39 (0.70–2.75)	4.93 (1.15–21.2)
Atopic dermatitis	**0.29 (0.12–0.68)**	1.10 (0.23–5.31)
Bronchial asthma	0.64 (0.25–1.67)	1.95 (0.23–16.1)
Number of family members (as one increases)	**0.68 (0.53–0.87)**	**0.72 (0.54–0.98)**
Working status (compared to not working)		
Part time	0.63 (0.31–1.28)	1.08 (0.44–2.64)
Full time	**2.14 (1.21–3.78)**	0.91 (0.43–1.94)
Education (compared to junior high and high schools)		
Junior and vocational colleges	1.64 (0.91–2.95)	2.03 (0.96–4.27)
University and graduate school	**3.75 (1.97–7.15)**	**3.02 (1.48–6.14)**
Annual household income (compared to less than JPY 4 million)		
JPY 4–6 million	1.73 (0.92–3.27)	1.05 (0.51–2.16)
JPY 6–8 million	**4.96 (2.34–10.5)**	1.90 (0.81–4.47)
JPY more than 8 million	**6.36 (2.79–14.5)**	**4.02 (1.37–11.7)**
Do not wish to answer	2.64 (0.97–7.23)	2.51 (0.51–12.4)

*OR, odds ratio; CI, confidence interval*.

*Bolds signifies *p* < 0.05*.

Multivariate analysis showed that household income remained significantly positively associated with varicella vaccination in Chiba city (Table 4). Children from a household with an income of JPY eight million or more were 6.55 [95% confidence interval (CI): 2.14–20.1] times more likely to receive varicella vaccination than children from a household income of less than JPY four million. However, in Nishitokyo city, household income was not associated with varicella vaccination in the multivariate analysis. No association between education and vaccine coverage was observed in either city. The *p*-value for interaction term between household income and city was 0.124, which might suggest that the subsidy was helpful in increasing vaccine coverage.

**Table 4:** **Multivariate analysis of association between varicella vaccination and variables of participants**.

	Chiba city OR (95% CI)	Nishitokyo city OR (95% CI)
Age of caregivers (compared to 20s)		
30s	**3.17 (1.56–6.43)**	1.77 (0.70–4.49)
40s	**4.65 (1.38–15.7)**	2.67 (0.69–10.4)
Female (compared to male)	0.84 (0.46–1.52)	1.13 (0.59–2.19)
Birth weight <2,500 g	**0.31 (0.10–0.94)**	1.91 (0.55–6.59)
Period of gestation <37 weeks	3.91 (0.73–21.1)	0.45 (0.14–1.41)
Past history		
Febrile seizure	0.93 (0.26–3.37)	0.61 (0.16–2.38)
Food allergy	2.54 (0.91–7.09)	3.56 (0.76–16.6)
Atopic dermatitis	**0.08 (0.02–0.26)**	1.13 (0.17–7.29)
Bronchial asthma	0.90 (0.24–3.44)	1.34 (0.14–12.8)
Number of family members (as one increases)	**0.55 (0.39–0.77)**	0.70 (0.49–1.01)
Working status (compared to not working)		
Part time	1.05 (0.44–2.55)	1.65 (0.53–5.14)
Full time	2.12 (0.96–4.67)	1.20 (0.46–3.09)
Education (compared to junior high and high schools)		
Junior and vocational colleges	1.05 (0.49–2.25)	1.31 (0.52–3.30)
University and graduate school	1.96 (0.83–4.67)	2.02 (0.81–5.06)
Annual household income (compared to less than JPY 4 million)		
JPY 4–6 million	1.89 (0.88–4.05)	1.03 (0.45–2.37)
JPY 6–8 million	**4.32 (1.71–10.9)**	1.42 (0.50–4.01)
More than JPY 8 million	**6.55 (2.14–20.1)**	2.98 (0.88–10.1)
Do not wish to answer	**4.27 (1.11–16.4)**	2.18 (0.39–12.1)

*OR, odds ratio; CI, confidence interval*.

*Bolds signifies *p* < 0.05*.

### Reasons for Not Vaccinating Their Children

We further asked the parents, who did not vaccinate their children, for the reason why they did not do so (Table 5). Few parents knew about the varicella vaccine (6.2 and 16.7% in Chiba and Nishitokyo cities, respectively), and 30.3% of parents in Chiba city regarded vaccine cost as a barrier compared to 11.9% in Nishitokyo city.

**Table 5:** **Reasons for parents not vaccinating children for varicella**.

	Chiba city (*N* = 145)	Nishitokyo city (*N* = 84)
Planning to vaccinate in future	25 (17.2%)	4 (4.8%)
Children are not expected to be vaccinated due to	26 (18.0%)	21 (25.0%)
Previous infection of varicella	24 (16.6%)	21 (25.0%)
Other medical conditions (such as post gamma-globulin therapy)	2 (1.4%)	0 (0%)
Have little knowledge on vaccines	9 (6.2%)	14 (16.7%)
Do not consider varicella to be a serious disease	4 (2.8%)	7 (8.3%)
Afraid of side effects of varicella vaccine	4 (2.8%)	6 (7.1%)
Consider varicella vaccine to be ineffective	2 (1.4%)	5 (6.0%)
Agree that there are barriers to vaccination	87 (60.0%)	33 (39.3%)
Cost of vaccines is too expensive	44 (30.3%)	10 (11.9%)
Have no time to take children for vaccinations	22 (15.2%)	9 (10.7%)
There are too many other vaccines	47 (32.4%)	20 (23.8%)
Effect of surroundings	19 (13.1%)	11 (13.1%)
Family doctors did not recommend the vaccine	15 (10.3%)	8 (9.5%)
Neighbors did not vaccinate their children either	6 (4.1%)	3 (3.6%)
Articles read on the internet advised not to vaccinate children	1 (0.7%)	0 (0%)
Other	6 (4.1%)	14 (16.7%)

## Discussion

### Impact of Subsidy on Varicella Vaccination

This study showed that household income was positively associated with varicella vaccine in an urban city where no vaccine subsidy was provided. This supports previous research that showed poverty to be a risk factor for underimmunization (6–9). Furthermore, the current study demonstrated the positive impact of the subsidy for varicella vaccination. This finding is consistent with a previous study that reported an increase in vaccine coverage from 8.0 to 17.2% in one rural city and 13.0 to 27.9% in another, after the introduction of a vaccine subsidy in 2007 (15).

The positive association observed between household income and vaccination in Chiba city (no subsidy) and the lack of such an association in Nishitokyo (partial subsidy) suggests that the subsidy was helpful in boosting vaccine coverage, regardless of household income. Moreover, about one-third of parents who did not vaccinate their children reported cost as a reason for not vaccinating in Chiba city (no subsidy), whereas only one-tenth of parents regarded cost as a barrier to vaccination in Nishitokyo (partial subsidy). Unlike a previous study conducted by Matsumura et al. in Japan (13), parents’ low knowledge on vaccination and concerns about possible adverse effects were not the major reasons for not vaccinating their children. One possible reason is the difference between vaccines in the two studies. The study by Matsumura et al. focused on the free measles-containing vaccine, whereas the present study focused on the varicella vaccine, which is not free. This might imply that a few parents might not vaccinate their children for varicella, even after the introduction of a complete subsidy. To address this issue, better promotion of the vaccine and building knowledge on varicella vaccination is still needed, even after introduction of the subsidy.

Factors other than socioeconomic ones were also associated with low vaccine coverage in multivariate analysis in Chiba city. These factors included younger age of caregivers, low birth weight, history of atopic dermatitis, and large number of family members. A previous study conducted in Japan also showed that the characteristics related to uncompleted measles vaccination were mother’s younger age and being a subsequent child (13). Little is known about the association of history of children with vaccine coverage in Japan, including low birth weight. The schedule of vaccine for children with low birth weight might be delayed because of long-term hospital stay and infection during infancy due to their vulnerability. Caregivers of children with atopic dermatitis might have concerns over drug allergies, which may be associated with caregiver’s reluctance in having their children vaccinated.

### Vaccine Coverage

The coverage of varicella vaccination in our study was 61.0% for infants aged 18 months in Chiba where no subsidy was provided, although a previous study showed that coverage of preschool children was around 30% (5). The reason for higher coverage in our study might be because our study setting comprised two clinics in urban areas of Greater Tokyo, where vaccine coverage is generally higher compared to rural areas of Japan. Although the Japanese government has promoted vaccines that are included in routine immunization programs, promotion of other vaccines depends on the local government. Therefore, urban cities with a bigger budget and a greater number of staff can disseminate those vaccines more easily, and this point might have contributed to the high vaccine coverage in our study.

### Limitations

First, our study did not directly show any changes in vaccine coverage caused by the introduction of the subsidy. However, Chiba and Nishitokyo cities are both commuting distance from Tokyo, and residents from both cities have common characteristics in terms of socioeconomic status. Thus, the difference in vaccine coverage is mainly attributable to the subsidy.

Second, our study could not examine the effect of the complete subsidy for the varicella vaccine. However, our study revealed the possibility that only a partial subsidy might improve vaccine coverage, which suggests that a complete subsidy would further improve vaccine coverage. Third, the method to recruit participants in the two cities and the age of children were different, which may affect the differential impact on the association between socioeconomic status and varicella vaccination. Further study is needed to investigate the impact of subsidy on vaccination, comparing the vaccination rate before and after introduction of subsidy, using children of the same age in the same city.

### Political Implications

Varicella vaccination is shown to be cost-effective in many studies (16), including a previous Japanese study (3) and the current study. Therefore, increasing vaccine coverage eventually reduces overall costs to the government, even if the government pays all costs of the vaccine. Moreover, subsidies for vaccination should be encouraged in order to remedy health inequality among children in countries where varicella vaccination is not included in routine immunization programs, which has received large attention in previous studies (17, 18). Children from households with a low income tend to have poorer health and achieve less academically, which reduces opportunities for a good job in future and, hence, less income. Therefore, subsidies for vaccines would also be helpful in tackling this cycle. Although subsidy is thought to be effective for boosting vaccine coverage, subsidy alone is not adequate because only half of the parents in Chiba city who answered the questionnaire stated that cost was a barrier for vaccination. To achieve desirable varicella vaccine coverage, not only monetary support but also the spread of correct knowledge on vaccine is important. Therefore, inclusion of varicella vaccine in national routine immunization program is essential.

## Conclusion

Varicella vaccine coverage of children aged 18 months was 61.0% in Chiba city where no subsidy was provided, whereas coverage of children aged 24–30 months was 73.3% in Nishitokyo. Multivariate analysis showed that household income was positively associated with varicella vaccination in Chiba city, while no association between household income and varicella vaccination was found in Nishitokyo city. These findings suggest that vaccine subsidy may be useful for children in low-income families. Thus, in countries where varicella vaccination is not included in routine immunization programs, introducing subsidies nationwide or routine immunization programs for varicella vaccination would be an important approach to eliminate inequality in vaccine coverage.

## Author Contributions

KN and TF conceived study, KN collected and analyzed data and wrote the first draft, and TF finalized the manuscript. KN and TF approved final version of manuscript.

## Conflict of Interest Statement

The authors declare that the research was conducted in the absence of any commercial or financial relationships that could be construed as a potential conflict of interest.
